# Endometrial CD56+ natural killer cells in women with recurrent implantation failure: An immunohistochemical study

**DOI:** 10.4274/tjod.galenos.2020.90359

**Published:** 2020-12-10

**Authors:** Gulchin Babayeva, Yunus Emre Purut, Burak Giray, Pembe Oltulu, Rabia Alakuş, Mehmet Cengiz Çolakoğlu

**Affiliations:** 1 Necmettin Erbakan University Meram Faculty of Medicine, Department of Obstetrics and Gynecology, Konya, Turkey; 2 Zeynep Kamil Women and Children Diseases Training and Research Hospital, Clinic of Obstetrics and Gynecology, İstanbul, Turkey; 3 Necmettin Erbakan University Meram Faculty of Medicine, Department of Pathology, Konya, Turkey

**Keywords:** CD56, immunohistochemistry, in vitro fertilization, recurrent implantation failure, uterine natural killer cells

## Abstract

**Objective:**

Implantation failure is a multifactorial problem of reproductive medicine. However, the mechanism of this process is still not fully understood. There is increasing evidence that these cases of recurrent implantation failure might have an immunologic background. Uterine natural killer (NK) cells provide immune-modulation at the interface between maternal decidua and the trophoblast. The aim of this study to evaluate whether there was a significant difference in the number of endometrial CD56+ NK between women with a history of recurrent implantation failure and women who had a live birth.

**Materials and Methods:**

Patients with a history of recurrent implantation failure were included in the study. Twenty-five women with a history of recurrent implantation failure were assigned to the case group, and 25 women who had one or more live births were assigned to the control group. Endometrial biopsies were obtained during the luteal phase on the 21^st^-24^th^ day of the menstrual cycle.

**Results:**

There was a statistically significant difference between the groups concerning the number of deliveries (p<0.001) and miscarriages (p<0.001). The mean number of uNK was 10.5±10.5 cells/mm^2^ in the case group and 19.2±11.2 cells/mm^2^ in the control group. There was a statistically significant difference between the two groups (p=0.003).

**Conclusion:**

Implantation failure is a multifactorial problem of reproductive medicine. The results of our study suggest that uterine NK play a role in the progress of normal pregnancy and reduced uterine NK cell numbers were associated with implantation failure.

**PRECIS:** We evaluated whether there was an effect of the number of endometrial CD56+ NK on women with a history of recurrent implantation failure.

## Introduction

Accomplished implantation of an eight-cell embryo into the endometrium is mandatory for reproduction. The rate of successful implantation of an embryo is approximately 30%^(1)^. Implantation failure is a multifactorial problem of reproductive medicine. Recurrent implantation failure (RIF) was defined as a failure of pregnancy after at least three previous assisted reproductive technique cycles, or implantation failure with a transfer of more than four embryos by the European Society of Human Reproduction and Embryology. A successful pregnancy requires synchronization between the trophoblasts and endometrium. However, the mechanism of this process is still not fully understood.

There is increasing evidence that these cases of RIF might have an immunologic background. The endometrium plays a role in implantation physiology via immune cells, cytokines, and chemokines^(2)^. Multiple kinds of immune cells are potentially involved in supporting immune tolerance during implantation and successful ongoing pregnancy^(3)^. Uterine natural killer (uNK) cells express their specific cell surface marker CD56, and differ from blood NK cells^(4)^. These uNK cells are the dominant leukocyte population (70-90% of uterine lymphocytes) in the decidua at the time of implantation and early placentation^(5)^. Although the function of uNK cells is uncertain, the regulation of uNK cells at the time of eight-cell embryo implantation is thought to feature in implantation. In a normal pregnancy, uNK cells provide immune-modulation at the interface between decidual tissue and trophoblast. We aimed to evaluate whether there was a significant difference in the count of endometrial CD56+ NK between women with RIF and women who had a live birth.

## Materials and Methods

### Participants and Study Design

Twenty-five women with RIF and 25 women who had one or more live births between January 2012 and December 2017 were included in the study. RIF was defined as the failure of good quality embryos to implant after at least 3 cycles of IVF. Women with positive for anti-phospholipid antibodies (ANA, anticardiolipin IgM and IgG, anti-DNA, antiphospholipid IgM and IgG), anti-toxoplasma IgM, and/or anti-rubella IgM were excluded from the study. Women with abnormal thyroid function tests results, anti-thrombin III deficiency, protein C or S deficiency, factor-V-Leiden mutation, prothrombin gene mutation, mutation of *MTHFR* *C677T* gene, and/or mutation of *MTHFR* *A1298C* gene were also excluded from the study. Hysterosalpingography was performed in all infertile patients before the procedure and there were no abnormal findings. Twenty-five women who had one or more live births were assigned to the control group. None of the 25 women in the control group received assisted reproduction treatment at any time. The local ethics committee approved the study (approval no: 20181146). The patients were informed orally and in writing.

### Collection of Tissue Samples and Histopathologic Examination

**Figure 1:**
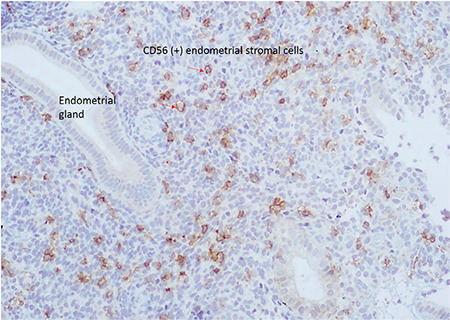
CD56+ cells were shown in the endometrial tissue

Endometrial biopsies were obtained during the luteal phase on the 21^st^-24^th^ day of the menstrual cycle using the Pipelle device and fixed in 10% formaldehyde. Formalin-fixed and paraffin-embedded tissue samples were incubated for 120-minutes with the CD 56 primary antibody (NCL-L-CD56-1B6, 1/200 dilution, Novocastra Laboratories Ltd.), 30 minutes with biotin (Dako LSAB System-HRP, Dako North America, Inc., K0690), and 30 minutes with streptavidin (Dako LSAB System-HRP, Dako North America, Inc., K0690), respectively. Aminoethylcarbazole chromogen was added for 15 minutes. Paraffin-embedded tissue samples were stained using Mayer’s hematoxylin. The same pathologist evaluated all samples using an Olympus BX53 microscope at 400x magnification. CD56+ cell counts were determined as cells/mm^2^ (Figure 1).

### Statistical Analysis

Data were analyzed using the SPSS software package (22.0, IBM SPSS Statistics for Windows; IBM Corp. Armonk, NY). Histogram, normality plots, and thre Shapiro-Wilk normality test were used to analyze data distribution. Descriptive statistics (mean, standard deviation, median, range, percentage) were used in the analysis of quantitative data. The chi-square (χ^2^) test or Fisher’s Exact test was used to analyze qualitative data. The Mann-Whitney U test was used in the analysis of quantitative data. Statistical significance was established at p<0.05.

## Results

Twenty-five women with RIF and 25 women as fertile controls were included in the study. The parental chromosomes were normal in all women. The mean ages of the case and control groups were 33.5±5.6 and 34.4±5.3 years, respectively. The two groups were similar in terms of age (p=0.224). There were statistically significant differences between the groups in terms of the number of deliveries (p<0.001) and miscarriages (p<0.001) (Table 1). The mean number of uNK was 10.5±10.5 cells/mm^2^ in the case group and 19.2±11.2 cells/mm^2^ in the control group. There was a statistically significant difference between the two groups in terms of uterine NK (p=0.003) (Table 1). There was a significant positive correlation between the number of uNK cells and the number of miscarriages (r=0.430) (p=0.002). There was also a significant positive correlation between the number of uNKs and the number of live birth (r=0.463) (p=0.001). Correlation coefficients of parity, live birth, miscarriages, and uNK of the patient group are shown in Table 2.

## Discussion

The endometrial leukocyte population consists of T-cells, macrophages, and natural killer cells. T-cells constitute 45% of leukocytes in the proliferative phase. Although their numbers remain constant throughout the cycle, their rate in proliferative phase is higher compared with other types of leukocytes. The most important leukocyte population in the endometrium comprises uNK cells. These lymphocytes contain the NK cell surface antigen CD56. During the implantation, large granular lymphocytes constitute 70-80% of the leukocyte population, and if conception occurs, their number increases more. UNK cells are particularly abundant in the uterus at the time of implantation and are in close contact with placental trophoblast cells. UNK cells have a major role in both implantation and the development of the placenta and vascularization. It limits the trophoblast invasion to decidua. In the absence of implantation, uNK cells undergo apoptosis and are therefore thought to play a role in the initiation of menstrual bleeding. UNK cells also secrete several growth factors involved in angiogenesis, such as VEGF, placental growth factor, and angiopoietin-2.

Giuliani et al.^(6)^ found that the number of endometrial CD56+ cells was not significantly different in women with recurrent pregnancy loss. By contrast, Quenby et al.^(7)^ demonstrated that the mean count of uNK cells was significantly higher in women with recurrent pregnancy loss than women with had a live birth history. Clifford et al.^(8)^ also described increased expression of uNK in 29 women with recurrent pregnancy loss. Similarly, in the current study, there was a positive correlation between the number of miscarriages and the amount of endometrial CD56+ cells. There was also a positive correlation between the number of live births and the number of endometrial CD56+ cells. Different etiologies except for reduced uNK, such as chromosomal abnormalities, could be a reason for miscarriage.

Sacks and Finkelstein^(9)^ found that uNK numbers increased dramatically from about 5% of stromal cells in the follicular and early luteal phases of the menstrual cycle to 30-40% of stromal cells in the mid and late luteal phases when implantation occurred. Gaynor and Colucci^(10)^ indicated that uNK numbers increased further to as much as 70% of stromal cells if implantation occurred. The current study demonstrated that there was a positive correlation between the number of live births and the number of endometrial CD56+ cells, and there was also a significantly reduced density of CD56+ cells in women with recurrent implantation failure. In contrast, Tuckerman et al.^(11)^ found that the high density of CD56+ cells in the endometrium of women with RIF was directly involved in implantation duration. Additionally, Santillan et al.^(12)^ found a higher density of endometrial CD56+ cells in women with RIF than in controls. They suggested that testing for endometrial NK cells might be helpful in women with idiopathic RIF during the luteal phase.

We acknowledge that the small sample size, retrospective nature, and lack of the chromosome analysis of the miscarriage tissues are the main limitations of the study. Increasing the number of patients and including other subgroups of CD 56+ cells to the study may help explain the mechanism.

## Conclusion

Implantation failure is a multifactorial problem of reproductive medicine. However, the mechanism of this process is still not fully understood. The results of our study suggest that uNKs play a role in the progress of normal pregnancy and reduced uNK cell numbers were associated with implantation failure. We believe that further studies will explain the role of these cells in the etiology.

## Figures and Tables

**Table 1:**
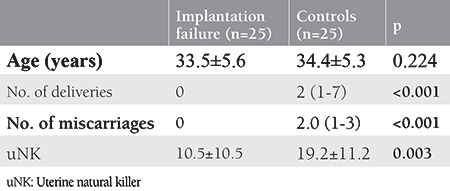
Patient demographics and comparison of the number of endometrial CD56+ NK between groups

**Table 2:**
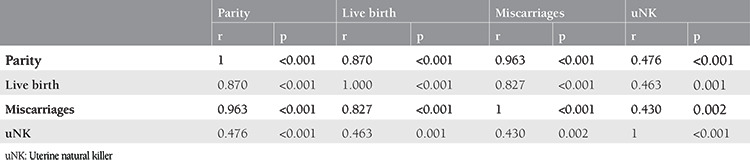
Correlation coefficients of parity, live births, miscarriages, and uNK of the patient group
